# Sleep disturbances as an early indicator of psychotic disorders in adolescents and young adults

**DOI:** 10.3389/fnsyn.2026.1949833

**Published:** 2026-09-04

**Authors:** Julia Jaromirska, Dominik Strzelecki, Magdalena Kotlicka-Antczak, Piotr Białasiewicz, Agata Gabryelska

**Affiliations:** 1 Department of Sleep Medicine and Metabolic Disorders, Medical University of Lodz, Lodz, Poland; 2 Department of Affective and Psychotic Disorders, Medical University of Lodz, Lodz, Poland; 3 Department of Child and Adolescent Psychiatry, Medical University of Lodz, Lodz, Poland

**Keywords:** clinical high risk for psychosis, early intervention, neurotransmission, sleep disturbances, synaptic dysregulation, teenagers, young adults

## Abstract

Sleep disturbances are increasingly recognized as clinically relevant markers in the early detection of psychotic disorders among adolescents and young adults. These disturbances, including insomnia, circadian rhythm disruptions, parasomnias, and fragmented sleep, affect up to 70% of individuals at clinical high risk for psychosis, rising to 80% in fully developed schizophrenia. This review synthesizes current evidence on the relationship between sleep problems and early psychotic symptoms, emphasizing their potential as observable and possibly modifiable indicators of illness onset. Neurobiological mechanisms suggest overlapping pathways for both sleep problems and the development of psychotic symptoms involving dopaminergic, serotonergic, and hormonal dysregulation, which are additionally exacerbated by psychological factors such as stress, substance use, and poor sleep hygiene. Screening methods, such as self-report questionnaires, actigraphy, and polysomnography, have demonstrated utility in identifying individuals at risk. Furthermore, sleep-focused interventions, particularly cognitive behavioral therapy, show promise in alleviating both sleep and psychiatric symptoms. Despite methodological limitations of available research, including heterogeneous study designs and short follow-ups, sleep assessment can represent a low-cost, scalable tool for early intervention. Future research should prioritize longitudinal, multimodal studies and explore personalized, sleep-centered preventive strategies. Furthermore, sleep disorders could be considered not only as early signs of developing psychosis but also as a potential therapeutic target in psychosis prevention.

## 1 Introduction

An average person spends one-third of their lifetime sleeping, with age being one of multiple factors affecting sleep duration and quality (Hertenstein et al., 2018). According to the American Academy of Sleep Medicine, sleep disturbances encompass a diverse and broad range of sleep-related problems, including circadian rhythm sleep–wake disorders, hypersomnias, insomnia, parasomnias, sleep-related breathing disorders, and sleep-related movement disorders. The estimated prevalence range of severe sleep problems is 7.8% in the adult population (Stubbs et al., 2018), whereas for children and adolescents, the global prevalence is not exactly specified. The problem in the pediatric population seems to be underdiagnosed and not heeded, which may have a detrimental effect on young individuals’ health. One of the limitations in detecting such conditions might be the disuse of detection methods, including sleep diaries, screening questionnaires such as the most popular Epworth Sleepiness Scale and Pittsburgh Sleep Quality Index (PSQI), polysomnography, or actigraphy. They act as quick and easy screening tests, but do not encompass all intricate or nonspecific sleep complaints. It has been found that sleep disturbances are associated with increased levels of systemic inflammatory markers (Cappuccio et al., 2010), plausibly increasing the risk of civilization diseases such as cardiovascular disease, cancer, or depression development. In addition, sleep disturbances in the form of an inadequate amount of sleep were proven to be associated with a greater risk of death (relative risk (RR): 1.12 for short- and RR: 1.30 for long-duration of habitual sleep) (Cappuccio et al., 2010). Sleep problems might be one of the first or the only symptoms of an emerging illness, as depicted in Parkinson’s disease (Kempfner et al., 2014), mood disorders (Pearson et al., 2023), thyroid disorders, or liver dysfunctions (Bruyneel and Sersté, 2018). A multidisciplinary approach is needed to determine whether the sleep disturbance occurs in isolation or as a symptom of a specific disease, which further may allow for the choice of an appropriate and effective treatment type.

According to the Diagnostic and Statistical Manual of Mental Disorders (DSM-5), psychotic disorders are defined by abnormalities in one or more of the 5 domains (delusions, hallucinations, disorganized speech, grossly disorganized or catatonic behavior, and negative symptoms), where at least one symptom must be delusions, hallucinations, or disorganized speech. The group includes diseases as follows: schizophrenia, schizophreniform disorder, schizoaffective disorder, delusional disorder, brief psychotic disorder, psychotic disorder due to another medical condition, substance/medication-induced psychotic disorder, and unspecified/other specified schizophrenia spectrum with other psychotic disorder (Substance Abuse and Mental Health Services Administration, 2016). The global prevalence of psychotic disorders is estimated at 0.39% for 12-month prevalence (Moreno-Küstner et al., 2018), whereas the median age of the onset of schizophrenia spectrum disorders is 25 years, and 47.8% of patients are diagnosed by this age (Solmi et al., 2022). Further, the peak age of onset is 20.5 years. Moreover, the duration of untreated psychosis is positively correlated with poor prognosis in first-episode psychotic diseases (Catalan et al., 2025). This data justifies the need to focus on detecting psychotic symptoms in the population of adolescents and young adults. The schizophrenia spectrum disorders frequently transition from previous short-lived psychotic episodes, and within a year, the risk is up to 19% (Provenzani et al., 2021).

Disturbed sleep is a common problem among adolescents and young adults, but especially for those who tend to have psychotic symptoms throughout life and with concomitant sleep disturbances, such as higher indications of poor sleep quality, total sleep time, sleep efficiency, sleep onset latency, and duration of wake after sleep onset in comparison to their peers (Dondé et al., 2022). Sleep disturbance prevalence in subsequent stages in the course of psychosis, starting from the clinical-high risk state, through early to chronic psychosis, is approximately 50% and does not differ significantly in each stage (Bagautdinova et al., 2023). Although the results regarding the link between the presence of sleep problems in clinical high risk for psychosis (CHR-P) and transition of this state into meeting full diagnostic criteria for psychosis remain inconsistent (Fekih-Romdhane et al., 2022a). It has been established that sleep disturbance is strongly and prospectively linked to increased psychotic symptoms observed in CHR-P (Zaks et al., 2022). It is also associated with worse psychosocial functioning (Nuzum et al., 2022) as well as with a shorter time to transition from CHR-P to psychosis (Nuzum et al., 2022).

The mentioned data strongly suggest a need for sleep disturbance observation in “high-risk” patients, which is also essential in terms of early detection and intervention in mental health. Understanding the intricate mechanisms behind sleep disturbance in pre/early psychotic patients may be a valuable insight that might allow for a better understanding of monitoring and intervention strategies. Therefore, this review aims to explore the potential role of sleep disturbances as early, observable biomarkers in adolescents and young adults at risk of developing psychotic disorders.

## 2 Adolescents’ and young adults’ sleep problems characteristics

The average sleep architecture in adolescents and young adults is characterized by insufficient sleep, which is often depicted by chronic sleep loss and subsequent daytime sleepiness resulting from sleep restriction (Owens et al., 2014). The most common causes include Chronotype change, use of stimulants, light exposure, the influence of school/academic year schedule, and psychosocial stress, which may trigger a psychotic episode in the presence of other risk factors.

### 2.1 Chronotypes and their influence on health

The sleep of children and adolescents differs significantly from that of adults. This, to a large extent, results from a hormonal shift, characterized by a reduction in nocturnal melatonin secretion (Overberg et al., 2022). Also, anatomical differences, including lower thickness of the cortex (especially the left orbital part of the inferior frontal gyrus and lower thickness of the mean curve of the right paracentral gyrus cortex) (Yang et al., 2024), contribute to the distinct sleep patterns in young individuals. Psychosocial factors depicted by a self-perpetuating circle, such as poor sleep quality, higher depression levels, and poor academic performance (Jongte and Trivedi, 2022), as well as smartphone addiction (Allouch et al., 2025) or use of addictive substances—alcohol, tobacco, cannabis, and other drugs (Leger et al., 2022) also play an important role in shaping sleep in adolescents and young adults.

Chronotype is an individual’s natural preference regarding the time of the day during which their functioning is optimal, and their energy is at its peak. An evening chronotype, also called late chronotype or commonly referred to as “night-owls,” is the most prevalent among young people as a well-known sign of the mentioned specific psychosocial and biological factors operating in this stage of life. Available data show that evening chronotype was associated with both a higher degree of mental health problems, such as more severe rule-breaking behavior and conduct problems, attention deficit/hyperactivity disorder (ADHD) symptoms, or affective problems (Merikanto et al., 2017), as well as with physical health issues, including higher BMI and higher frequency of non-healthy activities such as consuming unhealthy snacks, night-time caffeine consumption, or decreased daily intake of fruits and vegetables (Arora and Taheri, 2015). Those unhealthy habits are associated with early school start times, which exacerbate health problems in people with a late chronotype (Rodríguez Ferrante et al., 2024). Adolescents with an evening chronotype exhibit a higher risk of developing insomnia (Cheung et al., 2024) or poor sleep quality and daytime tiredness as a result of bedtime social networking site use in comparison to their peers with an early chronotype (Kortesoja et al., 2023). As mentioned above, chronic sleep deficiency is a risk factor contributing to emotional dysregulation. Furthermore, in the context of health, dysregulation also comprises emotional eating and body dissatisfaction (White et al., 2026), aggressive behaviors (Shi et al., 2023), or non-suicidal self-injury (Ennis et al., 2017). In addition, “owls” tend to spend more time on technology use at bedtime, aggravating those changes (Reardon et al., 2023).

### 2.2 Psychoactive substances

Psychoactive substances, which for this article can be briefly divided into 4 groups—stimulants, cannabinoids, opioids, and hallucinogens affect brain functioning to a large degree, inducing the desired result for the consumer. Alcohol, depending on the dose, may act as a stimulant or depressant (Tizabi et al., 2018). Temporary changes of such substances are based on the impact on neurotransmitters—mimicking or blocking them, depending on the substance. The broad choice of psychoactive substances includes widely accepted legal substances, such as coffee, energy drinks, nicotine, or morphine for medical purposes, and controversial drugs, prohibited in most countries, like lysergic acid diethylamide, cannabinoids, amphetamines, or 3,4-methylenedioxy-N-methamphetamine (MDMA). As they act directly on neuronal pathways, their effect may lead to neurotransmitter deregulation, and in this regard, they influence sleep subsequently. A cross-sectional study conducted in the United States among middle and high schoolers revealed that alcohol, amphetamine, cigarette, marijuana, and non-heroin narcotic use decreases significantly when used at least 7 h per frequency and inversely, pointing to a shared effect of sleep disruption in psychoactive substances (Terry-McElrath et al., 2016). In addition, psychoactive stimulants and antidepressants contributed to sleep paralysis more frequently than in young adults with no drug history (Gumbis et al., 2025).

#### 2.2.1 Stimulants

The use of widely distributed legal stimulants such as coffee, energy drinks, and cigarettes is frequent among adolescents and young adults and has a detrimental effect on sleep health. Especially, evening chronotype was associated with alcohol, caffeine, and nicotine use (Meneo et al., 2023). In Lodato et al., 10 mg/day of caffeine increased the odds of sleeping ≤8.5 h by 12% (Lodato et al., 2013). Likewise, energy drinks and alcohol consumption affect sleep, following shorter sleep duration and efficiency, longer sleep onset latency, and wake-up after sleep onset (Kaldenbach et al., 2024). It is associated with later average bedtime and wake time (Miller et al., 2021). Cigarette smoking has been linked to an increased risk of snoring and sleep apnea as well as decreased sleep quality (da Silva e Silva et al., 2022).

Amphetamines and their structural analogs, taken both legally—e.g., as a form of treatment for ADHD—and illegally, impact sleep. Amphetamine, used as a recreational drug, is taken by 4.05% adolescents globally, with prevalence of abuse among boys 5.14% and among girls 2.34%, and the most popular region of amphetamine recreational use is Africa (Son et al., 2025). To date, there is still a lack of research identifying sleep changes potentially associated with amphetamine use disorder. However, the topic of using amphetamines as medication has been widely described. Methylphenidate, a stimulant that acts as a norepinephrine-dopamine reuptake inhibitor, has a bilateral effect on sleep, according to the literature, improving sleep quality and decreasing nighttime awakenings. However, it alters sleep patterns, resulting in a decrease in total rapid eye movement (REM) and non-REM (NREM) sleep, as well as an increase in sleep onset latency (Jaromirska et al., 2025). In comparison to methylphenidate, amphetamine treatment in ADHD adolescents did not induce later sleep onset time on medicated days, but was suggested to worsen sleep efficiency on medicated days compared to those with methylphenidate implemented (Wiggs et al., 2024). In addition, according to the meta-analysis by Punja et al., including 2,429 children/adolescents (RR 3.80) (Punja et al., 2016), the use of amphetamines in ADHD treatment was associated with insomnia. Amphetamine-type stimulants used by young patients with affective or psychotic spectrum illnesses were associated with poorer sleep quality over the course of the disease. The key factors involved were the occurrence of depressive symptoms, daily amphetamine-type stimulant use, and socio-occupational functioning (Crouse et al., 2018).

MDMA, commonly known as ecstasy, is an amphetamine derivative, a connectogen leading to the triggering of a massive release of serotonin and oxytocin. Interestingly, in terms of sleep, the recreational use of MDMA has been associated with a higher prevalence of obstructive sleep apnea (McCann et al., 2009). This phenomenon may be partially explained by the detrimental effect on brain serotonin in young adults, independent of age, gender, race, and obesity status.

#### 2.2.2 Cannabinoids

Cannabinoids represent one of the most commonly used substances among adolescents in high-income countries, with an estimated prevalence of abuse of 9.45% (Son et al., 2025). The sleep effect of cannabinoids stratifies with age, changes across the young population (with the increased risk of long/short sleep), mid-life adults (with the increased risk of long sleep), and the elderly (with the increased risk of short sleep) (Gonzalez et al., 2023). Cannabinoid use in adolescents was linked with poorer sleep outcomes in 48% of reported cases (Amaral et al., 2023). Cannabinoid withdrawal has been associated with exacerbated sleep disturbances when no treatment for addiction is implemented (Sullivan et al., 2022). What partially stands in opposition to the effect of recreational drugs, medicinal cannabinoid substances have been studied for promoting sleep in a variety of clinical cases with positive results, managing sleep disturbances in the form of insomnia or nightmares (Belendiuk et al., 2015). Particularly, non-cannabidiol medications showed improvement in sleep quality (da Silva et al., 2025).

### 2.3 Light exposure

The effect of exacerbated bedtime light exposure affects both adolescents and young adults, independently of the chronotype (Estevan et al., 2022). Various kinds of light exposure, including outdoor artificial light at night (Wang et al., 2022) and late evening electric light (Louzada et al., 2024), and above all, the use of light-emitting devices in the evening, often overlapping with time spent in bed (Van der Maren et al., 2018), affect sleep patterns in humans. A more and more prevalent problem connected with light exposure during bedtime may be smartphone usage (Exelmans and Van den Bulck, 2016). Young people tend to spend the majority of their time being connected to the internet, constantly maintaining social networks with their peers. As shown in Goel et al., smartphone use time among undergraduate students correlated positively with the PSQI score, pointing to poorer sleep quality (*β* = 0.142, *p* = 0.040, *r*^2^ = 0.027) (Goel et al., 2023). Irregular bedtime and nocturnal phone usage (e-mail exchange, calling after lights-out) were connected to a higher chance of being involved in school bullying (Tochigi et al., 2012). Such aggressive behaviors may be the result of a sleep pattern change, affecting subsequent daytime functioning. That is why there is a need for parental control in using electronic devices during bedtime (Pieters et al., 2014). As a social phenomenon influencing both sleep hygiene and mental health, social networking site use in young people decreases sleep quality and increases the feeling of loneliness in young adults (Moretta et al., 2023). In addition, loneliness was found to be an independent factor influencing sleep quality, especially among individuals subjected to victimization in adolescence or maltreatment in childhood (Matthews et al., 2017).

### 2.4 The influence of school and academic year schedule

Another emerging factor in shaping sleep patterns is the school and university timetable, which forces students to adjust to a morning schedule for 5 days of the week, and then allows them to choose their sleep routine on free days or during vacation. As observed by Estevan et al., in adolescent subjects, sleep onset and offset significantly extended on weekends in comparison to school days, with sleep duration 2 h longer. However, recovery sleep-free days did not eliminate changes induced by the previous 5-day sleep restriction (Zitting et al., 2018). As reported by Jiang et al., 5-day sleep restriction (from 8 to 6 h of sleep) was associated with impaired reaction time in working memory tasks among adolescents without a significant change in subjective sleepiness score, and increased subjective sleepiness score among young adults without a substantial change in reaction time in working memory tasks (Jiang et al., 2011). Sleep restriction affects older adults as well, but research has shown that younger adults are more prone to those changes (Zitting et al., 2018). In the group of 14-18-year-old schoolers, mood significantly worsens after 1 day of sleep deprivation, with a higher vulnerability for females, with depressive and anxiety symptoms being the most pronounced Sleep restriction in the form of 2.5 h sleep shortening over 3 weeks in young people in comparison to their peers with healthy sleep, resulted in the aggravation of unfavorable emotional states including tension, anxiety, anger, hostility, confusion, and fatigue. Shorter sleep time and sleep deprivation in healthy adolescents were linked to a 55% increase in mood deficits, as a meta-analysis by Short et al. demonstrated (Short et al., 2020). Whereas night sleep restriction decreases cognitive performance, napping attenuates the decline but does not eliminate it (Zitting et al., 2018). A changeable sleep pattern was also examined in the student group, leading to poor sleep quality in over 60% of the participants, with the main problems being delayed weekend bedtime and risetime and sleep–wakefulness states altered with prescription, over-the-counter, and recreational psychoactive drugs. Importantly, stress and tension accounted for 24% of the variance in the PSQI score in a multiple regression analysis (Lund et al., 2010). Different university activities may also impact sleep, as was demonstrated in college athletes, with 42.4% experiencing poor sleep quality and 39.1% experiencing inadequate sleep time. Young people engaged in university sport activities tend to have more disrupted sleep in comparison to those without such activities. It may be a result of the strict university schedule, which is filled with training and competitions, and the necessity of joining compulsory classes. In addition, the highest mean level of daytime sleepiness was reported in first and second-year students (Mah et al., 2018).

As mentioned above, psychological distress in the form of academic pressure and social stress, together with lifestyle factors such as technology use, stimulant use, and poor sleep hygiene, as well as biological factors, including circadian phase delay and hormonal shift, affect adolescents’ and young adults’ sleep, leading to decreased sleep duration, sleep quality, as well as subsequent wakefulness quality, making young individuals more prone to develop other health disorders.

## 3 Looking for the early signs of psychotic disorders in youth

More than 23% of young people are thought to be experiencing at least one psychotic experience, depending on the population (university students are more prone in comparison to high school students), country (young people living in the United States are more prone in comparison to United Kingdom, Japan or Australia), and exposure to COVID-19 (young people who did not experience the disease are more prone in comparison to those who experienced it) (Fekih-Romdhane et al., 2022b). While a first psychotic episode may signal underlying mental illness, it can also be triggered by environmental factors, such as substance use, psychological distress, or physical illness (Radua et al., 2018). Another issue that requires clinical attention is recognizing patients with clinical high risk for psychosis (CHR-P) and the need to monitor their mental state, as the conversion rate to a threshold psychotic disorder is 17.5% (Raballo et al., 2022). The symptoms include attenuated psychotic symptoms, brief, limited, intermittent psychotic symptoms, and vulnerability manifestations, including genetic risk and/or schizotypal personality traits associated with functional decline. In Chen et al., where CHR-P symptoms were divided into 5 groups, including sleep disturbances, terror, indecision, gradual loss of sadness and despair, and experiences of self-denial and uncertainty, sleep disturbance was further divided into the most prevalent 3 categories: unhealthy sleep habits, difficulties in falling asleep, and permanent daytime fatigue (Chen et al., 2025). However, as the research had a qualitative design, no predictive values were obtained. It is also essential to diagnose CHR-P with caution, as transient psychotic symptoms appear in almost 8% of adolescents (Kelleher et al., 2012).

Schizophrenia is the most prevalent diagnosis among primary psychotic disorders, affecting approximately 1% of the population, with a genetic predisposition heritability of between 60 and 85% (Escudero and Johnstone, 2014). The early signs of schizophrenia cannot distinguish it from other psychotic diseases, including brief psychotic disorder and schizophreniform disorder, which have a better prognosis. Therefore, any high-risk symptoms should be monitored. Besides the commonly known risk factors and prodromal symptoms, modern medicine focuses on finding early endophenotype markers of schizophrenia. Numerous hallmarks may relate to sleep parameters as well. Blood-based predictors such as cytokines, complement proteins, genome, transcriptome, proteome, and hormonal traits are being searched for; however, without any clinical use yet (Byrne et al., 2023). However, the most promising included fatty acid depletion, VEGF dysregulation, leukocyte miRNAs, glutathione, or SNPs of cytokine genes—interleukin −1, −2, −6, −10 and their ratio (Mondelli et al., 2023). A new approach also concerns electrophysiological markers, searching for early indications of psychosis in electroencephalography, which may detect subjects at a high risk or in early stages, but with no prominent clinical symptoms (Perrottelli et al., 2021). Observed EEG abnormalities may vary or change depending on previous sleep activity. For the same reason, neuroimaging biomarkers are being searched for, including changes in both structural and functional magnetic resonance imaging. The latest growing field in this research area includes digital phenotyping and artificial intelligence, analyzing passively and actively collected data via sensors, surveys, etc., in order to map changes in the pattern of human behavior (Benoit et al., 2020).

Substance/medication-induced psychotic disorder, although not a primary psychotic disorder, but caused by substance intoxication/withdrawal, can convert into more serious burden with the expected transition from substance/medication-induced psychotic disorder into schizophrenia is 25% with the most important factors triggering transition including abuse of cannabis (34%), hallucinogens (26%), amphetamines (22%), opioids (12%), alcohol (10%), and sedatives (9%) (Murrie et al., 2019). Another key factors that predispose to conversion include younger age (statistically significant for men) and repeated emergency admissions related to substance-induced psychosis (statistically significant for men and women) (Rognli et al., 2023). In most cases, the medical intervention for substance-induced psychotic disorder is based on obtaining and maintaining abstinence from addiction; however, vulnerable subjects might need broader observation and more substantial interventions.

The onset of psychotic disorders frequently occurs at a young age; therefore, it is essential to emphasize the role of early detection and observation of the high-risk group for disease conversion. There is also a need to search for early markers of psychotic disorder development.

## 4 Sleep disturbances in the prodromal phase and early stage of psychotic disease

As mentioned before, sleep abnormalities are a common feature in the prodromal stage of psychosis as well as during the first episode of the disease, affecting almost 70% of the high-risk group (Cohen et al., 2024), aggravating up to 80% in fully developed schizophrenia (Cohrs, 2008). Therefore, sleep changes appear to be a promising risk factor for psychotic disease development (Lunsford-Avery et al., 2013). An important aspect of the relationship between sleep disturbances and psychosis might be the age of disease onset. Among adolescents and young adults, the neuronal pathways are still not fully developed, which may contribute to more detrimental consequences of the ongoing psychotic process. Moreover, subtle changes may be mistakenly ascribed to teenage rebellion or adolescence (Tiffin and Kelleher, 2025). That is why noticing nuanced indicators, especially among the teenage/young adult group, could be beneficial.

Poe et al. showed that in individuals with CHR-P, the main sleep disturbances included sleep pattern disruption (related to greater intensity of positive symptoms and worse overall functioning), day-night reversal (related to greater intensity of positive symptoms), and daytime fatigue (related to greater intensity of negative symptoms) (Poe et al., 2017). A study by Lunsford-Avery et al. revealed that increased movements during sleep were present in Ultra High-Risk for Psychosis (UHR-P), positively correlating with positive symptoms observed in this group of patients (Lunsford-Avery et al., 2015). Moreover, several objective measures of sleep disturbance predicted the longitudinal course of symptoms over 12 months in the UHR group. Another study from the same group showed that sleep patterns in UHR-P individuals are often characterized by increased sleep onset latency and fragmented continuity; in addition, sleep disturbances negatively correlated with the bilateral thalamus volume in the “at risk” group. In Zaks et al., although sleep pattern could not predict conversion to disease, disturbed sleep correlated with a more severe course of the CHR-P symptoms (both positive and negative). Depression accounted for half of the association between sleep and symptoms. The treatment of sleep problems appears to be a useful tool in ameliorating symptoms present in the CHR-P state (Poe et al., 2017).

Circadian rhythm disruption depicted by circadian rhythm fragmentation and low daily activity, was shown to be associated with more severe psychotic symptoms among CHR-P young individuals. In a study by Castro et al., CHR-P participants demonstrated more fragmented and less synchronized circadian rhythms with lower amplitude of motor activity and concomitant worse sleep quality, longer nap duration, shorter wake time, higher total sleep time, and shorter activity duration (Castro et al., 2015). Additionally, Purple et al. found a positive correlation between rapid-eye movement sleep duration and motor skill memory among the CHR-P group but not controls (Purple et al., 2020). The authors suggest that sufficient sleep, specifically longer REM phase duration, appears to improve motor performance among CHR-P who exhibit subthreshold psychotic symptoms. Together with desynchronization from the light/dark cycle, lower daily activity, and more fragmented rhythms contributed to the prediction of psychosis symptoms severity, such as greater positive and negative symptoms, and psychosocial impairment at 1-year follow-up (Lunsford-Avery et al., 2017).

Other sleep disorders associated with psychotic symptomatology include parasomnias. The presence of frequent nightmares in childhood has been associated with an increased likelihood of later psychotic experience despite gender, family adversity, emotional/behavioral problems, intelligence quotient, and neurologic problems, as observed in Fisher et al.; in addition, night terrors, or sleepwalking, were shown to be more common among children with subsequent psychotic experiences (Fisher et al., 2014).

The presence of insomnia in healthy young adults is a risk factor for psychotic-like episodes. As reported by Cohen et al., 25% of CHR-P patients experience terminal insomnia, but without association with conversion to a psychosis disorder (Cohen et al., 2024). As research has already shown, treating insomnia with psychological intervention had a significant positive effect on sleep, mood, and functioning in the CHR-P and early psychosis group (Waite et al., 2018).

Sleep disturbance is linked to negative daytime mood and can predict enhanced psychotic symptoms on the following day in CHR-P individuals. Although the presence of specific sleep disorders did not correlate with conversion to clinically significant psychotic disorders, their recognition and treatment might be associated with clinical benefits, improving symptom severity and perhaps preventing or delaying transition to a psychotic disorder (Bradley et al., 2018). It has been established that sleep disturbances are associated with a shorter time in conversion to psychosis (Nuzum et al., 2022). No evidence to date suggests that parasomnias alone can predict psychosis onset; however, they may serve as clinical “red flags” when combined with other prodromal symptoms. Table 1 summarizes the research data on sleep disorders in individuals at risk for psychosis. However, the primary findings synthesized in Table 1 should be interpreted with caution due to several methodological and statistical limitations across the reviewed studies. Objective actigraphy and neuroimaging investigations frequently rely on small sample sizes (*n* = 20–36) without formal power calculations, increasing the risk of type II errors, whereas larger multicenter studies (*n* > 680) offer greater statistical power but rely solely on subjective measures. Furthermore, many studies suffer from incomplete statistical reporting and lack explicit corrections for multiple testing, raising the potential for type I false positives across unadjusted univariate models. Finally, cross-study synthesis is constrained by sample heterogeneity, including wide age ranges (12–30 years) and inconsistent control for key clinical confounders such as psychotropic medication use and comorbid affective symptoms.

**Table 1:** Sleep problems among CHR-P patients.

Sleep problem	Group of patients	Assessment type	Assessed value in CHR/ARMS	References
Daytime fatigue	194 CHR-P, 66 HC; aged 13–30	Structured interview for prodromal syndromes G1	Prevalence: 28.9% vs. 3% (*F* = 20.30, *p* < 0.01) Association: prediction of negative symptoms (*B* = 3.12, *p* = 0.02)	Poe et al. (2017)
Sleep pattern disruption			Prevalence: 17.5% vs. 0% (*F* = 13.92, *p* < 0.01) Association: prediction of positive symptoms (*B* = 3.37, *p* < 0.01) and negative (*B* = 4.48, *p* < 0.01) symptoms, lower functioning (*B* = −4.46, *p* < 0.01)	
Day night reversal			Prevalence: 11.9% vs. 0% (*F* = 8.86, *p* < 0.01) Association: prediction of positive (*B* = 3.05, *p* < 0.01) and negative (*B* = 5.54, *p* < 0.01) symptoms	
Reduced sleep efficiency	36 CHR-P, 31 HC; aged 12–21	Actigraphy, Sleep/activity diary, PSQI, BDI	ANCOVA *F*(3,62) = 4.33, *p* = 0.008 CHR < HC	Lunsford-Avery et al. (2015)
Increased total movement during sleep			ANCOVA *F*(3,62) = 5.75, *p* = 0.01 CHR > HC	
Increased wake after sleep onset			ANCOVA *F*(3,62) = 5.03, *p* = 0.01 CHR > HC	
Increased sleep dysfunction (increased latency, decreased efficiency, reduced quality) with decreased thalamus volume	33 CHR-P, 33 HC, aged 12–21	MRI, PSQI	For left thalamus: increased latency *F*(7,14) = 6.68, *p* = 0.01, decreased efficiency *F*(7,13) = 4.32. *p* = 0.011, reduced quality *F*(7,14) = 13.91, *p* = 0.001; For right thalamus: increased latency *F*(7,14) = 3.55, *p* = 0.020, decreased efficiency *F*(7,13) = 5.02, *p* = 0.006, reduced quality *F*(7,14) = 6.56, *p* = 0.002	Lunsford-Avery et al. (2013)
Longer nap duration	20 ARMS, 20 HC, aged 13–27	Actigraphy, ESS, PSQI, MEQ	χ^2^ = 6.54, df = 2, *p* = 0.038 ARMS>HC	Castro et al. (2015)
Lower autocorrelation functions			χ^2^ = 9.66, df = 2, *p* = 0.008 ARMS<HC	
Lower interdaily stability			χ^2^ = 8.40, df = 2, *p* = 0.015 ARMS<HC	
Higher intradaily variability			χ^2^ = 9.65, df = 2, *p* = 0.008 ARMS>HC	
Worse subjective sleep quality	688 CHR-P, 94 HC, aged 12–30	PSQI	Mean: for CHR non-converters 1.36 [0.78], for CHR converters 1.41 [0.88], for controls: 0.82 [0.53] ANOVA: *F*(2,779) = 21.78, *p* < 0.001	Zaks et al. (2022)
Circadian rhythm fragmentation	34 CHR-P, 32 HC, aged 12–21	Actigraphy, sleep diary	Mean: for CHR − 0.29 [0.11], for controls −0.26 [0.07] ANCOVA: *F*(3,61) = 3.65, *p* = 0.017	Lunsford-Avery et al. (2017)
Longer onset of nocturnal rest			Mean: for CHR 1587.35 [108.44], for controls 1542.95 [115.06] ANCOVA: *F*(3,61) = 3.32, *p* = 0.025	
Terminal insomnia	688 CHR-P aged 12–30	SOPS, PSQI	Prevalence: 25% Prediction: conversion to psychosis over longitudinal follow-up (HR = 1.64, 95% Cl [1.12, 2.41], *p* = 0.011)	Cohen et al. (2024)
Sleep disturbance			Prevalence: 68.9% Association: correlation with greater severity of negative symptoms and lower global functioning (*p* < 0.001)	

ARMS, At-Risk Mental States; BDI, Beck Depression Inventory; CHR-P, Clinical High Risk Patients; ESS, Epworth Sleepiness Scale; HC, Healthy Control; MEQ, Morningness-Eveningness Questionnaire; PSQI, Pittsburgh Sleep Quality Index; SOPS, Scale of Prodromal Symptoms.

## 5 Neurobiological and psychological mechanisms

As described above, sleep changes are linked to CHR-P, suggesting that they may share a common pathway leading to the onset of psychotic disorders. Understanding the underlying mechanisms of this relationship is essential for effective treatment and potentially useful for reducing the risk of conversion to a psychotic illness. Shared neurobiological pathways, as well as the bilateral relationship between sleep disturbances and other CHR-P symptoms, concomitant stress, emotional regulation, and cognitive processing, might expose a new target in tracking down and observing individuals at risk. A meaningful role for both sleep and psychiatric deficits may be played by the thalamus and hypothalamus, especially the suprachiasmatic nucleus (Trbovic, 2010).

### 5.1 Neurotransmission

Alterations in neurotransmission characterize both sleep disorders and psychotic symptoms. Particular neurotransmitters, with dopamine and serotonin at the forefront, seem to be crucial to the shared pathophysiology of sleep and psychosis (Monti and Monti, 2004). Main information derives from psychosis treatment and its impact on sleep disturbances. Psychosis treatment with classical antipsychotic drugs ameliorated sleep disturbances, pointing to a role in psychosis development or maintenance of dopaminergic receptor abnormality. Dorsun et al. reported that risperidone treatment in individuals with schizophrenia significantly ameliorated sleep dysfunction in comparison to a typical antipsychotic drug (Dursun et al., 1999). However, the results are not consistent, as in a study by Yamashita et al., the only difference in the risperidone group was a longer slow-wave sleep period (Yamashita et al., 2002). The second atypical antipsychotic drug, amisulpride, which binds dopamine D2 and D3 receptors, antagonized the detrimental effect of sleep deprivation on EEG record, psychomotor, and cognitive performance (Patat et al., 1999). Olanzapine has been shown to increase total sleep time and slow-wave sleep, with corresponding increases in theta wave activity in healthy females (Lindberg et al., 2002). 5-HT2 blockage induced by atypical antipsychotic drugs correlated with the amelioration of negative symptoms among psychotic patients (Yamashita et al., 2004). Serotonin receptors’ functioning may have a dual, simultaneous result in decreased sleep duration and understimulation of opioid receptors (Ionov, 2010). Initial increased dopaminergic activity, which is believed to occur in psychotic events, may secondarily affect cholinergic activity, which in turn affects sleep architecture by reducing REM latency. According to this, treatment with dopamine mimetics may induce psychosis, but only with concomitant cholinergic system disruption (Bosboom et al., 2003). As reported by Perry et al., blockage of muscarinic receptors induced hallucinations, whereas cholinergic activation in REM sleep induced dreaming (Perry and Perry, 1995). In postmortem studies of schizophrenic brains, the diminished muscarinic M1 and M4 receptors were found with a reduced number of nicotine receptors, especially in the hippocampal area (Crook et al., 2000, 2001), in which activity is particularly connected with the REM phase (Del Rio-Bermudez and Blumberg, 2022).

### 5.2 Synaptic-level change

Sleep regulation and the neurodevelopmental trajectory toward psychosis are deeply connected with cellular processes such as synaptic stability, dendritic spine density, and structural neuroplasticity. During adolescence and early adulthood—a crucial window for both brain maturation and the peak onset of psychotic disorder—the delicate balance between synaptic formation and elimination becomes particularly vulnerable (Pawlak et al., 2025). Understanding how synaptic-level alterations affect sleep architecture and may facilitate early psychotic progression is key to bridging cellular pathology with clinical manifestations in youth at CHR-P.

#### 5.2.1 Mismatch negativity

One of the neuronal hallmarks present in CHR-P patients includes the reduction of mismatch negativity (MMN) event-related potential, indicating N-methyl-D-aspartate receptor (NMDAR)-dependent auditory echoic memory and short-term plasticity (Umbricht and Krljesb, 2005). Translational and genetic evidence links glutamatergic synapse pathology directly to MMN; for instance, loss-of-function mutations in schizophrenia risk genes encoding NMDAR subunits directly diminish MMN responses and cortical network synchronization (Herzog et al., 2023). As the EEG, MRI, and saliva examination of 303 CHR-P and blood examination of a subset of 57 of them revealed, there is a close association between MMN amplitudes and salivary cortisol and pro-inflammatory cytokines—the more deficient MMN amplitudes, the higher peripheral biomarker levels—but only in CHR-P converters within the next 2 years (Hamilton et al., 2025). In addition, MMN amplitudes correlate with reduced cortical gray matter volume in the CHR-P converters sample—together with chronic low-grade inflammation and elevated stress markers, they reflect localized synaptic destabilization, accelerated dendritic spine elimination, and impaired neuroplasticity during early disease trajectories via NMDAR hypofunction, which may lead to an excess of weak synaptic connections (Hamilton et al., 2025).

The plausible association of such findings with sleep disturbance may arise because MMN exhibits a direct neurophysiological relationship with sleep architecture and micro-arousal mechanisms (Atienza et al., 2002). Daytime pre-attentive sensory processing (indexed by MMN) and nighttime sleep oscillations depend critically on intact NMDAR signaling and thalamocortical dendritic spine density; excessive synaptic pruning during adolescence has been proposed as a mechanism that could affect both domains. Clinically, such changes may be reflected by reduced slow-wave sleep duration, altered REM sleep dynamics, and diminished sleep spindle density (Bagautdinova et al., 2023). Both MMN generation during wakefulness and SWS/spindle oscillations during NREM sleep rely heavily on the functional integrity of thalamocortical networks and NMDAR-mediated glutamatergic signaling (Parras et al., 2020; Liu et al., 2026). Furthermore, daytime manifestations of sleep disruption in CHR-P cohorts, such as severe daytime fatigue and circadian rest-activity fragmentation, may be explained by blunted MMN (Sallinen and Lyytinen, 1997; Gumenyuk et al., 2010), but direct research is still lacking. Because SWS and sleep spindles are critical for nocturnal synaptic renormalization and memory consolidation, their deficit combined with NMDAR hypofunction may contribute to a pathophysiological feedback loop: underlying synaptic alterations may attenuate MMN and disrupt sleep architecture, while fragmented sleep may further exacerbate daytime pre-attentive sensory deficits.

#### 5.2.2 Dendritic spine density

Beyond electrophysiological indices of plasticity, structural remodeling at the dendritic level plays a pivotal role in modulating both sleep architecture and psychosis vulnerability in youth. Accelerated reductions in cortical gray matter (GM) volume, as observed through structural MRI, may help differentiate CHR-P who are likely to develop the disorder from those who are not. Such changes may depict diminished spine density and abnormal dendritic arborization, leading to neurodegenerative lesions (Pasternak et al., 2012). Longitudinal imaging reveals that microstructural gray matter disruption (indexed by increased interstitial free water) predates macrostructural volume loss across prefrontal, temporal, parietal, and occipital lobes in individuals at CHR-P (Cho et al., 2024). Crucially, CHR-P converters exhibit an accelerated trajectory of increased interstitial free water elevation and gray matter volume reduction over time compared to non-converters (Cho et al., 2024).

Interestingly, the exact relationship between clinical sleep disruption and these structural alterations appears to operate at the microstructural and functional, rather than macrostructural, level. In a prospective study including 507 CHR-P and 81 healthy controls, sleep disturbances (measured via PSQI) significantly predicted an increased risk of conversion to psychosis (OR = 1.059, *p* = 0.04). However, no associations were observed between subjective sleep quality and macrostructural gray matter volume in the thalamus, hippocampus, or cerebellum among CHR-P nor control group (Wezenberg et al., 2025). This negative finding suggests that sleep disruption and gray matter alterations may be related to mechanisms distinct from gross macrostructural volume loss, potentially involving microstructural synaptic dysregulation, NMDA receptor hypofunction, and neuroinflammatory processes.

### 5.3 Hormones

One of the hormones relating to connections between sleep and psychosis through dopaminergic signaling might be prolactin, as a direct positive relationship was found between prolactin and REM latency among patients with non-affective psychosis (Appelberg et al., 2002). The plausible mechanism involves a prolactin increase in response to dopamine inhibition, which in turn prolongs REM latency.

**Figure 1:**
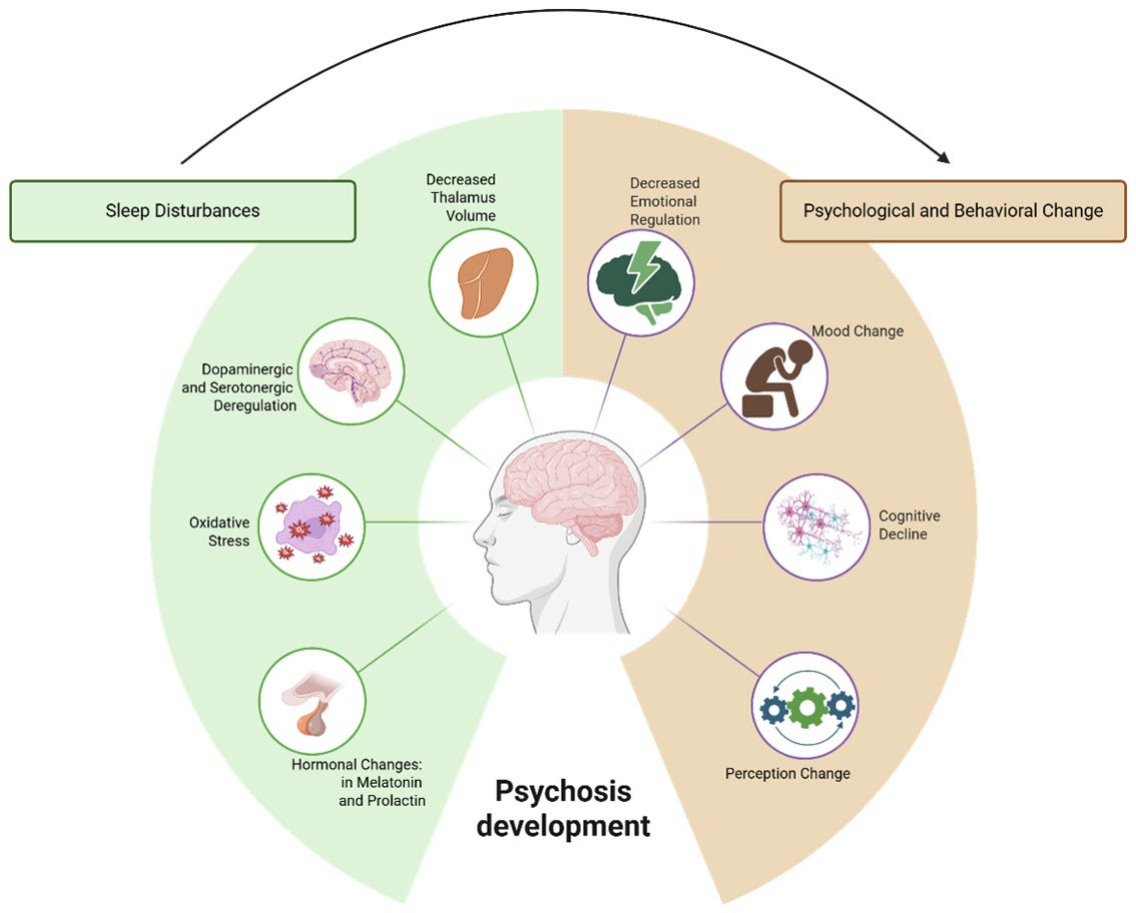
Molecular mechanisms connecting sleep and psychosis risk.

The second, more probable hormone involved is melatonin, although the plausible relationship between melatonin and psychosis has not been widely investigated. Both CHR-P and first-episode patients were reported to have a reduction in pineal volume compared to controls (Takahashi et al., 2022); in addition, the presence of melatonin cysts revealed in MRI was associated with severe positive psychotic symptomatology. In animal studies, melatonin appeared to act as a neuroprotective antioxidant in the rat brain, which served as a model for schizophrenia development (Zararsiz et al., 2007). In addition, decreased melatonin blood levels in schizophrenic patients correlated with lower cognitive performance and evening chronotype (Sahbaz et al., 2019). In the study by Igwe and Brigo, melatonin intake together with atypical antipsychotics improved the metabolic side effects in bipolar but not schizophrenic patients (Igwe and Brigo, 2018). In comparison, in Modabbernia et al., short-term melatonin treatment together with olanzapine alleviated metabolic symptoms, including weight increase, abdominal obesity, and high triglyceride levels (Modabbernia et al., 2014). As metabolic syndrome is connected to a higher possibility of developing sleep disturbances, the association between melatonin imbalance and the influence of psychotic drugs seems to be worth highlighting. The interactions between sleep disturbances, psychosis development, and psychological/behavioral change are illustrated in Figure 1.

## 6 Clinical implications

Integrating subjective and objective sleep data may allow clinicians to monitor disease progression and implement timely, targeted interventions.

### 6.1 Screening tools

Sleep disturbances in CHR-P subjects should be recognized as clinically relevant and considered in routine assessment, given their potential impact on clinical outcomes. In addition, sleep tracking offers the potential for early identification of individuals with CHR-P, making it a valuable component of a screening tool for this condition. A first step into a more personalized approach is observation, identification, and recognition of the present and reported sleep problems by the patient.

Most of the studies on the topic used self-report questionnaires as screening tools, such as the PSQI (Zaks et al., 2022) or Insomnia Severity Index (ISI) (Waite et al., 2023), which are quick and easy to administer, not only in research, but also in everyday practice. High scores on self-report screening tools (PSQI>5 or ISI > 14) indicate subjective sleep dissatisfaction and hyperarousal, which correlate with worsening positive symptom trajectories and lower levels of anterior cingulate glutamate (Korenic et al., 2020); these findings may support consideration of cognitive-behavioral therapy (CBT) for insomnia or sleep-focused psychological support. Sleep-focused interventions include sleep hygiene routines, psychological therapy with an emphasis on previously mentioned CBT, and biological treatment with the use of melatonin at the forefront, tailored to the patient’s individual needs. An additional benefit might be gained by antipsychotic treatment with drugs improving sleep profile.

On objective monitoring, actigraphic markers such as reduced interdaily stability, elevated intradaily variability, or prolonged sleep onset latency serve as continuous indicators of circadian desynchronization and fragmented rest. As a systematic review showed, lower motor activity and longer sleep duration were associated with more severe negative symptoms in patients, whereas less organized patterns of motor activity and poorer sleep quality were associated with more pronounced positive symptoms, impaired cognitive functioning in terms of attention and processing speed, longer illness duration, higher antipsychotic medication doses, and lower quality of life (Wee et al., 2019). Clinically, marked actigraphic disruptions might signal a heightened risk for short-term symptom exacerbation, which may warrant closer clinical monitoring and consideration of sleep–wake hygiene measures, circadian interventions, or adjustment of daily social/academic routines.

Finally, polysomnographic alterations, specifically reduced slow-wave sleep and shortened REM latency, reflect underlying trans-thalamic cortico-cortical dysregulation (Vukadinovic, 2011). Particularly reduced sleep spindle activity has been associated with the development of psychotic experiences (Kammerer et al., 2024). When identified, these electrophysiological markers can indicate a more advanced risk state, prompting closer longitudinal monitoring for threshold psychotic symptoms, family involvement, and a lower threshold for initiating non-pharmacological or tailored psychiatric interventions.

### 6.2 CBT approaches

CBT has demonstrated efficacy in improving sleep and functional outcomes in patients with schizophrenia and related psychotic conditions; however, research is scarce in the CHR-P area (Chiu et al., 2018). In a study by Waite et al. CBT therapy for insomnia named SleepWell, providing knowledge and skills for improving sleep, was applied to young (mean age 16.9, range 14–23) CHR-P individuals. The intervention consisted of 8 one-hour sessions over a 12-week period of time with additional contact between sessions, if needed, and focused on improving three sleep domains: sleep pressure, circadian rhythm, and hyperarousal. Main activities included promoting daytime activities with a daily activity time point, creating morning routines, using light–dark exposure, and nighttime relaxation. Participants of the study reported a great value of several aspects of the therapy, such as recognition of the problem, a multidisciplinary approach to a solution, and obtaining personalized help. The intervention improved sleep variables, psychiatric symptoms, and social functioning, suggesting that it may be a feasible and effective approach for young CHR-P patients with insomnia (Waite et al., 2023). Improving sleep hygiene knowledge and providing tools needed for its implementation seem to be the most crucial factors in psychological intervention to increase sleep quality and, via this, to improve composite mental health. The influence of other therapeutic approaches proposed for CHR-P, like family-focused therapy or integrated psychological therapy has not been explored in terms of their influence on sleep quality (Minichino et al., 2025).

### 6.3 Pharmacological interventions

When CBT fails to achieve clinical improvement in CHR-P individuals, pharmacological treatment with atypical antipsychotics may be considered (Poletti et al., 2024). As mentioned before, atypical antipsychotic drugs had better sleep outcomes in comparison to typical ones. However, side effects for young people constitute a major source of subjective distress, resulting in negative general well-being and non-compliance (Schimmelmann et al., 2005). Moreover, available data do not provide a rationale for antipsychotic use to prevent the conversion of CHR-P into first-episode psychosis (Di Lisi et al., 2024; Minichino et al., 2025).

### 6.4 Other interventions

Other, less researched, but in need of further understanding, treatments include electroconvulsive therapy, which was found to improve not only first-episode psychosis itself, but also polysomnographic measures with a positive change in REM latency, REM density, and sleep efficiency (Zhang et al., 2012); however, the use of the method, especially in “at-risk” subjects rises many ethical questions and definitely requires further research.

Another possible treatment, melatonin use among CHR-P patients or patients in the early stage of psychotic disease, has not been researched to date. However, the efficacy of melatonin as a circadian rhythm treatment in schizophrenia and other psychotic disorders is well-established; its use in CHR-P has not been assessed so far.

The use of polyunsaturated fatty acids, such as omega-3 fatty acids, may be beneficial, as research shows that their intake reduces insomnia and improves sleep quality (Ortega et al., 2025), while, when it comes to psychosis, some studies determined positive effects of omega-3 s on CHR-P, but without any statistical significance in conversion in meta-analysis (Susai et al., 2022). Despite contradictory research results and low statistical power, omega-3 s are included as one of the therapies for alleviation of CHR-P symptoms, including cognitive deficits (Chen et al., 2024; Winter-Van Rossum et al., 2025). That is why their influence might be used in relieving sleep problems in CHR-P as well; however, this hypothesis has not been researched yet.

**Figure 2:**
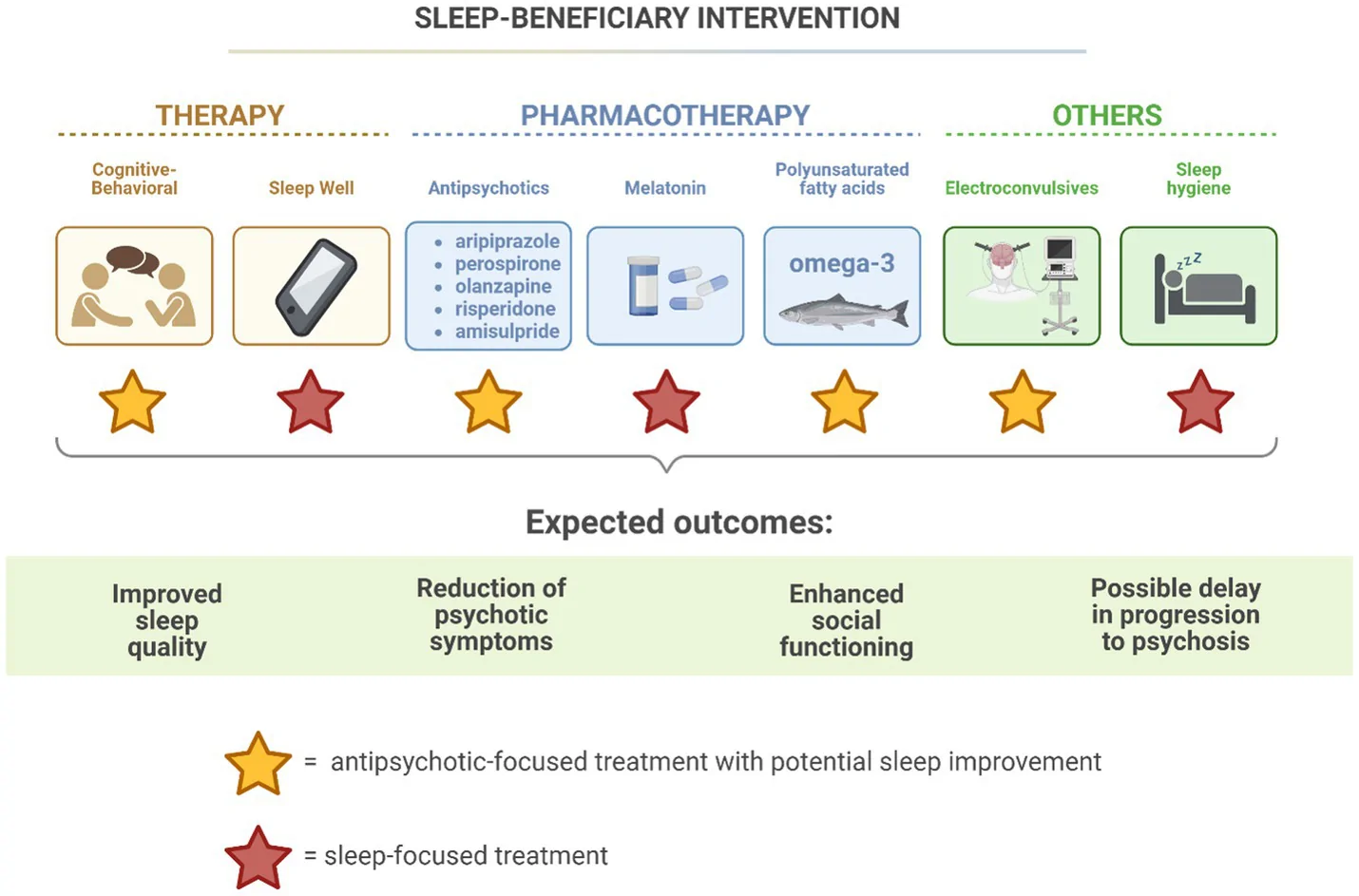
Sleep-beneficiary interventions in high-risk of psychosis or early psychosis patients.

Contemporary guidelines for treating the CHR-P condition recommend specific psychological interventions, mostly in the form of CBT, with secondary antipsychotic drug use, if the symptoms persist (Poletti et al., 2024). For patients with minor sleep problems, the basic treatment of the condition itself could help improve sleep quality. However, if the background therapy did not show expected improvement, special sleep-focused treatment acting through neurobiological and psychological pathways described above might be beneficial. All of the mentioned interventions, both conventional CHR-P treatment with positive sleep effect (marked with gold stars) and typical sleep-focused interventions (marked with red stars), are depicted in Figure 2.

## 7 Methodological considerations and limitations

Awareness of sleep disturbances among adolescents and young adults with CHR-P or early-stage psychotic disorders is increasing; however, the problem is still being frequently underplayed. As patients’ experiences reveal, the most difficult part in finding proper help for their mental health problems was accessing specialized services offering comprehensive help concerning both primary psychosis prevention and sleep treatment, as one inevitably affects the other (Waite et al., 2025). Nowadays, early intervention services mostly use reliable tools for diagnosing CHR-P. However, most of these tools do not refer to sleep disturbances, which seem to be an essential part of the clinical picture of early psychosis. Hence, there is a need for determining and implementing easy-to-use methods for estimating sleep problems in young individuals who experience early psychotic symptoms. Screening tests used so far in young populations include self-report questionnaires, actigraphy, polysomnography, sleep logs, and consumer wearables (Zhang et al., 2012; Castro et al., 2015; Hennig et al., 2020; Lahti et al., 2021; Zaks et al., 2022). Recommended sleep questionnaires comprise: the PSQI to assess overall sleep quality, the ISI to evaluate the severity of insomnia symptoms, and the ESS to measure daytime sleepiness.

As far as research regarding sleep disturbances in early phases of psychosis development is concerned, several methodological limitations should be noted. Most research focuses on CHR-P, not first-episode psychosis (FEP), as well as on adolescents, not young adults, which narrows the scope of discussion. In addition, the majority of studies are based on a single method of measuring sleep quality and disturbances. To date, only one program has been evaluated as a tailored intervention, which was the SleepWell psychological intervention in the form of sleep-oriented CBT. There was no other study concerning the influence of CHR-P/FEP treatment on sleep and vice versa. Future studies should prioritize multi-modal sleep assessment and longitudinal designs capturing both pre- and post-treatment outcomes.

## 8 Future directions

The bidirectional relationship between sleep disturbances and psychosis development remains incompletely understood. The most prone groups include adolescents and young adults, as psychotic disease onset frequently occurs during those periods of life. Therefore, monitoring clinical features of pre-psychosis and concomitant symptoms, which increase the likelihood of psychotic episode occurrence, such as sleep disturbances, appears to be fundamental. More reliable research is needed with larger groups of participants and follow-ups longer than 12 months. In order to find which sleep features in particular are significantly connected with psychosis onset and its course, more detailed sleep monitoring should be implemented in CHR-P and FEP patients. This part entails creating and testing new, cheaper, more convenient, and widespread technological tools for sleep tracking. Lastly, new preventive strategies in at-risk populations should be developed and implemented clinically, as sleep disturbances, among other factors, can contribute to psychosis development. Strategies should also be more comprehensive and personalized depending on the patient’s problem, needs, and resources.

## 9 Conclusion

This review highlights sleep disturbances as a clinically relevant, modifiable marker in the early detection of psychosis. Given the evidence linking sleep alterations with CHR-P states and early-stage psychosis, the incorporation of sleep assessments into the routine evaluation of patients reporting psychotic symptoms may facilitate detection and improve clinical outcomes. Personalized, sleep-focused interventions, especially CBT, show promise in ameliorating both sleep and psychiatric symptoms. Further research is needed to clarify the pathophysiological underpinnings and optimize treatment strategies.
